# Physiological Adaptation to Simultaneous Chronic Exposure to High-Fat Diet and Dichlorodipheniletylhene (DDE) in *Wistar* Rat Testis

**DOI:** 10.3390/cells8050443

**Published:** 2019-05-10

**Authors:** Vincenzo Migliaccio, Raffaella Sica, Rosaria Scudiero, Palma Simoniello, Rosalba Putti, Lillà Lionetti

**Affiliations:** 1 Department of Biology, University of Naples, Federico II, 80126 Naples, Italy; vincenzo.migliaccio@unina.it (V.M.); raffaella.sica@unina.it (R.Si.); rosaria.scudiero@unina.it (R.Sc.); rosalba.putti@unina.it (R.P.); 2 Department of Chemistry and Biology “Adolfo Zambelli”, University of Salerno, 84084 Fisciano, Italy; 3 Department of Science and Technologies, University of Naples, Parthenope, 80133 Naples, Italy; palma.simoniello@uniparthenope.it

**Keywords:** environmental pollutants, hyperlipidic diet, oxidative stress, apoptosis, cell proliferation, androgen receptor

## Abstract

Environmental chemicals can be introduced by consuming contaminated foods. The environmental chemical dichlorodiphenyldichloroethylene (DDE), a persistent metabolite of dichlorodiphenyltrichloroethane (DDT), can affect spermatogenesis. Our study aims to evaluate, by using spectrophotometric analyses, western blot, and immunohistochemistry, the adaptive responses in testis of adult rats treated with a non-toxic dose of DDE, alone or in association with a high-fat diet (HFD). Four experimental groups were performed: N (normal diet); D (HFD); D + DDE (HFD + DDE); N + DDE (normal diet + DDE). D group showed a reduction in antioxidant capacity, and increases in lipid peroxidation, apoptosis, and proliferation associated with morphological impairment. A reduction in androgen receptor (AR) and serum testosterone levels were also found. DDE-treated groups exhibited higher lipid peroxidation levels compared to N and D, associated with pronounced defect in antioxidant capacity, apoptosis, cellular proliferation, as well as with tissue damage. Moreover, decreases in AR and serum testosterone levels were found in DDE-treated groups vs. N and D. In conclusion, HFD and DDE produced cellular stress leading to antioxidant impairment, apoptosis, and decreases in AR and serum testosterone levels associated with tissue damage. Cellular proliferation could be used as an adaptation to counterbalance the occurred damage, maintaining a pool of tubules that follow physiological maturation.

## 1. Introduction

In the last few decades, experimental proof and data analysis of scientific studies have correlated male infertility both with environmental pollution and nutritional choices [1,2]. In fact, a variety of chemicals introduced with contaminated foods [3,4] and the excess of energy intake [5], determine the alterations in hormonal levels, sperm parameters, and production, and induce infertility [6]. Regarding excessive food intake, especially saturated fatty acids, Jensen and co-workers [7] indicated that they cause changes in semen quality and alteration in spermatogenesis with reduced levels of sperm concentration in humans. Moreover, experimental data showed negative correlation between a diet rich in saturated fatty acids and sperm fatty acid profiles associated with other sperm parameters’ alterations [8]. In fact, it has been shown that in human spermatozoa, elevated consumption of saturated fatty acids and a low consumption of omega-3 levels, in particular docosahexaenoic acid (DHA), induced alterations in lipid composition of sperm cell membrane and consequently in its structural integrity affecting its important role in fertilization [8]. In addition to an excess of saturated fat intake, many hydrophobic compounds derived from human activities, such as insecticides like dichlorodiphenyltrichloroethane (DDT) and its metabolites, can be absorbed in the body’s cells through contaminated food [9,10]. Many studies demonstrated that these environmental contaminants used in the last few decades alter the male reproductive system by functioning as antiandrogenic compounds, leading, in the most serious cases, to male infertility [11,12,13,14,15]. These hydrophobic substances can accumulate in the environmental matrix and undergo bioaccumulation and biomagnification phenomena in the food chain [16], particularly in animal fat tissue. In general, these molecules are very stable and have a long persistent time [17]. DDT is a synthetic insecticide belonging to the polychlorinated biphenyls (PCB), environmental pollutants widely utilized in the past years in various industrial and commercial applications. The effects of PCB metabolites included carcinogenicity, neurotoxicity, and reproductive impairment [18]. DDT was extensively used to control the main parasite vectors, such as mosquitoes and phlebotomies [19]. It was banned in Europe and North America in the 1970s because it constituted a threat to wildlife, particularly to birds [20] and mammals, with potential deleterious effects also on human health [21]; as a result, the levels in biota have decreased worldwide [22,23]. Moreover, DDT and its metabolites can reach remote world regions where it is no longer used, or has never been used, via atmospheric transport [24,25]. In fact, DDT is still used nowadays as an insecticide for the control of parasites in countries where malaria is a major public health concern [26]. It has been shown that DDT is a powerful endocrine disruptor, by acting as androgen receptor antagonist [27] and decreasing levels of testosterone in both serum and testis [28] with dramatic consequences on spermatogenesis and fertility. Its major persistent metabolite, 1,1-dichloro-2,2 bis (p-chlorophenyl) ethylene (alternatively known as p,p’-DDE or DDE), remains in the environment and has been detected in the serum, plasma, and tissues of humans despite a widespread ban of DDT [29,30,31]. DDE retains the reproductive toxicity, in fact many abnormalities in sexual development in rats and wildlife have been associated with exposure to p,p’-DDE [27,32], as well the inhibition of androgen binding to the androgen receptor [27,33], and increased rate of testicular apoptosis [34]. DDE exposure seems to be generally associated with increased reactive oxygen species (ROS) production and lipid peroxidation, confirming oxidative stress as an important component in DDE-induced toxicity [35]. Moreover, both DDT and DDE alter Sertoli cell–cell communications, acting on membrane stability and function [36]. Most studies have been carried out on cultured cells or administering moderately high doses of DDE (50–100 mg/kg body weight per day) intraperitoneally [37,38,39] or by gavage [40]. Hence, we decided to investigate the effect of a chronic lower oral DDE dose (10mg/kg b.w. per day), alone or in combination with a high-fat diet administered for 28 days. Although this dose is still very high, if compared to the environmental exposure levels, it has been demonstrated that after similar administration, DDE concentration measured in rat tissues was comparable to those detected in the human population [41]. 

The goal of this paper was to clarify whether a high-fat diet and DDE, alone or in combination, may induce male reproductive dysfunction or adaptive responses. To unveil how a high-fat diet and DDE affect testicular functions, we analyzed: (a) Oxidative damage in terms of tissular malondialdehyde (MDA) accumulation, as one of the lipid peroxidation’s products [42]; (b) quantitative variations of the activities of principal enzymes implicated in the control of ROS accumulation: Superoxide dismutase (SOD) and glutathione peroxidase (GPx) that act during spermatogenesis [43,44]; (c) analysis of p53 levels and variations in the degree of apoptosis by using western blotting of the pro-apoptotic protein Bcl-2-associated X protein (also known as BAX) and immunohistochemical stain of the active caspase 3; (d) cellular proliferation, in terms of the proliferating cell nuclear antigen (PCNA) protein content, used to counterbalance cellular death; (e) variations of the metallothioneins (MTs) in terms of gene expression and protein content; (f) histological alterations of testis; (g) serum testosterone levels and variation of androgen receptor (AR) content. Our results seemed to indicate that an excess of fatty acids intake, as well as xenobiotic species introduced with contaminated foods, can negatively act on testicular function by inducing metabolic and morphological changes, which can ultimately lead to infertility. However, an increase in cellular proliferation was found to be stimulated. This mechanism was probably activated to support spermatogenesis as an adaptation to induced-stress conditions.

## 2. Materials and Methods

### 2.1. Experimental Model

Experiments were carried out on sexually mature male *Wistar* rats aged 2 months (Charles River Italia, Calco, Como, Italy), kept one per cage in a temperature-controlled room at 24 °C with a 12 h light–dark cycle. The study was performed in strict accordance with the criteria established by the National Institutes of Health. The Committee on the Ethics of Animal Experiments of the University of Naples Federico II approved the protocol (Permit Number: 2012/0024690).

At the start of the study, after 7 days of acclimatization, 32 rats were randomly allotted into four experimental groups composed of 8 rats for each group. Two groups received a normal laboratory diet (standard control diet PF1915, HTD.06416 Harlan Laboratories, 15.47 KJ/g, 10% fat J/J, lard 20 g/kg; fatty acid profile (% of total fat): 29% saturated, 37% monounsaturated, 34% polyunsaturated) and were called N and N + DDE. The other two groups received a high-fat diet (PF1916, HTD.06415 Harlan Laboratories, 19.23 KJ/g, 45% fat J/J, lard 195 g/Kg,; fatty acid profile (% of total fat): 36% saturated, 47% monounsaturated, 17% polyunsaturated) and were called D and D + DDE. Rats from N + DDE and D + DDE groups were exposed to DDE (10 mg/kg body mass in corn oil) via oral administration every day for 28 days. DDE dose was chosen on the basis of previous data showing that the oral administration of such doses for 6 weeks did not affect physical development and sexual maturation in pubertal rats, or serum metabolic parameters in male adult rats [45]. The period of treatment of 28 days was chosen since it is a period of time that usually induced the earlier metabolic alterations due to the high-fat diet [46] and moreover, it has been shown that the administration of the chosen dose of DDE for 28 days did not give rise to any overt signs of toxicity in male rats [47]. Animals from N and D groups received only corn oil in the same manner of DDE-treated animals. After the treatment period, the rats were anesthetized by an intraperitoneal injection of Zoletil (40 mg/kg body weight) and euthanized by decapitation. One testis for each animal was immediately frozen in liquid nitrogen and stored at −80 °C for subsequent molecular analyses. The other testis was removed, washed in cold ice NaCl 0.9%, fixed in Bouin’fluid for 12 h at room temperature, dehydrated in ethanol, embedded in paraplast, and sectioned to 5 µm with a microtome.

### 2.2. Lipid Peroxidation

The effect of the treatment on the testicle oxidative damage for lipids was assessed by a quantitative analysis of malondialdehyde (MDA) as one of the final products of the lipid peroxidation reaction using a thiobarbituric acid reactive substances (TBARS) assay kit (Cayman Chemical Company, No.10009055). The amount of MDA in each sample group was analyzed and the result was expressed as nmol MDA per mg of protein. 

### 2.3. SOD and GPx Activity Assay

SOD and GPx activities were measured using two different kits provided by the Cayman Chemical Company: Superoxide Dismutase Assay Kit (No.706002) and Glutathione Peroxidase Assay kit (No.703102). A small piece of testis from each animal was processed according to the assay kit, homogenized in the cold homogenization buffer, and centrifuged. Then, the supernatant obtained was used for the analysis. SOD activity was expressed in enzymatic units per liter (U/L), whereas GPx activity was expressed as nmol/min per mg of protein.

### 2.4. Electrophoresis and Western Blot Analysis

Extract of total testicular proteins was obtained using RIPA Buffer (150 mM NaCl, 50 mM Tris, 1% NP-40, 0.25% sodium deoxycholate, 0.1% SDS, pH 8.0) and a cocktail of protease inhibitors (Sigma Aldrich). 150 mg of tissue were homogenate in 1 mL of RIPA buffer by using a polytron (KINEMATICA Polytron Model PT10-35 GT/PT 3100D Homogenizer, Fisher Scientific), and centrifuged at 12,000× *g* for 15 min. The pellet was discarded, and the supernatant used for analyses. Then, 30 µg of proteins, quantified by using the Hartree method, were electrophoresed on 13% SDS-polyacrylamide gels under denaturing conditions by using the Laemmli procedure [48]. The samples were electrophoresed with a pre-stained protein marker (Color Burst Electrophoresis Marker m.w. 8000–220,000 Da, Sigma Aldrich). After running electrophoresis, the proteins were transferred onto nitrocellulose membranes (Immobilon-P, Millipore) at 350 mA for 1 h. Membranes were blocked in blocking buffer solution composed of TBS-Tween solution (1 × TBS + 1% Tween-20, Sigma Aldrich) + 5% milk for 1 h at room temperature. Then, membranes were incubated overnight (O.N.) at 4 °C with the primary antibodies of interest diluted in TBS-Tween + 2% milk. The proteins analyzed by western blotting were tested using specific primary antibodies: p53 (rabbit polyclonal antibody, Cell Signaling (9282), 1:500); BAX (rabbit polyclonal antibody, sc-526, 1:200); PCNA (rabbit monoclonal antibody, Cell Signaling (D3H8P), 1:500); MT (mouse monoclonal antibody, ThermoFisher Scientific, (MA1-25479), 1:500); AR (rabbit polyclonal antibody, Abcam, ab133273); and ß-actin (mouse monoclonal antibody, sc-70319, 1:200) used as total loading control guide. The second day, membranes were washed in TBS-Tween buffer and incubated in TBS-Tween + 5% milk for 1 h at room temperature, with the secondary antibody labeled with horseradish peroxidase (donkey-anti rabbit, IgG-HRP: sc-2313, 1:5000 or goat-anti mouse, IgG-HRP: sc-2005, 1:5000). Again, membranes were washed in TBS-Tween solution and finally used to detect the relative densities of the immunoreactive bands by using the C-DiGit Chemiluminescent Western Blot Scanner (LI-COR). Signal optical density (OD) of bands for each protein analyzed were normalized using the density of ß-actin immunoreactive bands in each sample. The data obtained were graphed as fold changes of normalized OD in the treated rats vs. controls using GraphPad software.

### 2.5. Immunohistochemistry

Several sections were processed by immunohistochemical reaction using the Novolink Polymer Detection System (Leica Biosystems). After antigen retrieval and quenching of endogenous peroxidase, the sections were incubated O.N. at 4 °C with anti-cleaved Caspase 3 (rabbit monoclonal antibody, Cell Signaling (D175) (5A1E), diluted 1:300 in cold ice PBS) to detect apoptotic cells. The second day, slides were washed in PBS to eliminate the unbound primary antibody and the immunostaining was performed according to the Novolink Polymer Detection System protocol, using the 3,3′-diaminobenzidine (DAB) as chromogen. Nuclei were counterstained with Hematoxylin staining. The reaction specificity was tested: 1) By omitting the primary antibody and incubating the sections with either non-immune serum (1:10 in PBS) or 1% BSA; 2) by absorbing the primary antiserum with its specific peptide (10 nmol/mL of appropriately diluted Ab), O.N. at 4 °C. Images of sections, dehydrated in ethanol and mounted with cover slip, were acquired using a Zeiss Axioskop microscope fitted with a TV camera.

### 2.6. Quantitative Real-Time PCR Analysis

Total mRNA was extracted according to the Tri-Reagent protocol. RNA quality was checked by electrophoresis on 2% agarose gel stained with ethidium bromide and measuring the optical density 260/280 nm ratio. QuantiTect Reverse Transcription Kit (Qiagen) was used for the removal of genomic DNA contamination and for the subsequent cDNA synthesis. Approximately 1 μg of total RNA was used, according to the kit’s protocol. For the MT expression analysis, the Applied Biosystem 7500 Real-Time PCR System and the Power SYBR Green PCR Master Mix (Life Technologies) were used by following the procedures recommended by the manufacturer. Each amplification reaction contained 12 μL of real-time PCR Master Mix, 1 μL of forward and 1 μL of reverse primers (10 μM each), 2 μL of cDNA diluted 1:1 and 4 μL of nuclease free water, for a final volume of 20 μL. Nuclease-free water for the replacement of cDNA template was used as a negative control. Primer sequences were designed using Primer Express software (Applied Biosystems). A single pair of specific primers for both *Rattus norvegicus* MT1, MT2 isoforms, and β-actin (used as control) were designed as described in literature [49,50]. The PCR was performed under the following conditions: Holding stage of 95 °C per 10 min; cycling stage (45 cycles): 95 °C × 10 s, 60 °C × 10 s, 72 °C × 10 s; melting stage: 95 °C × 5 s, 65 °C × 1 min, 95 °C × 30 s, 40 °C × 30 s. The melting curve analysis of PCR products was performed in order to ensure gene-specific amplification. Changes in the gene expression relative to the different samples were calculated according to the standard 2^−ΔΔCt^ method [51].

### 2.7. Histological Analysis

Testis sections were spread on slides and stained with haematoxylin and eosin to observe the morphological status of testis. The slides were examined under a Zeiss Axioskop light microscope and the digital images were acquired using a TV camera attached to the microscope.

### 2.8. Serum Testosterone Levels

Testosterone levels were quantified by using a standard ELISA kit from Enzo Life Sciences Cat. Nos. ADI-900-065.

### 2.9. Statistical Analysis

All data were shown as mean ± standard deviation (SD). Statistical analyses were carried out with Graph Pad software. Differences between mean values obtained from control and treated groups were analyzed by one-way analysis of variance (ANOVA) followed by Bonferroni Post-hoc test. The differences were considered significant when *p* value was <0.05. Two-way ANOVA with Bonferroni Post-hoc test was also performed.

## 3. Results

### 3.1. High-Fat Diet and DDE Induce Testicular Lipid Peroxidation

**Figure 1:**
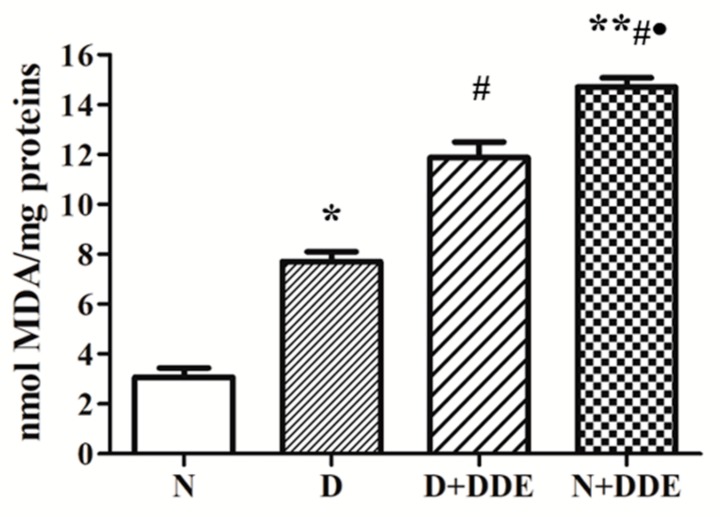
Analysis of malondialdehyde (MDA) accumulation in rat testis homogenate. The results were graphically represented as mean ± standard deviation in each group. One-way ANOVA analysis showed significant differences between treated and control rats: * *p* < 0.05 vs. N; ** *p* < 0.01 vs. N; **#** *p* < 0.05 vs. D; • *p* < 0.05 vs. D + DDE. Two-way ANOVA analysis indicated very significant diet effect, extremely significant DDE effect, and extremely significant interaction (Bonferroni Post-hoc test: *p* < 0.001 D vs. N, *p* < 0.001 D + DDE vs. N + DDE).

The amount of MDA in treated animals vs. N was graphically reported as nmol of MDA per mg of protein (Figure 1).

The analyses showed a significative fold increase of MDA in D vs. N (2.51 ± 0.25). The highest levels of MDA were found in presence of the pesticide. In the D + DDE group, a significant increase was found vs. D (1.54 ± 0.16). Moreover, in N + DDE, significantly higher levels of MDA were measured vs. N (4.78 ± 0.24), D (1.91 ± 0.09), and D+DDE (1.24 ± 0.06).

### 3.2. Modulation of Antioxidant Enzymes Activities: SOD and GPx

The activities of SOD and GPx showed an impairment of antioxidant capacity due to the different treatments.

**Figure 2:**
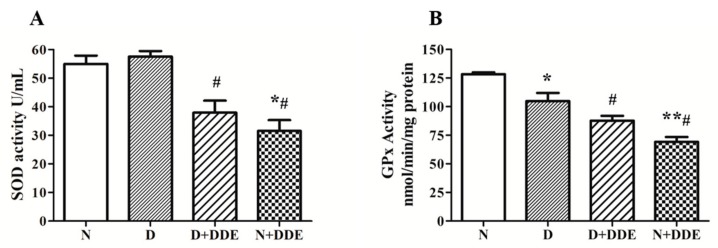
Activity of total superoxide dismutase (SOD) (**A**) and total glutathione peroxidase (GPx) (**B**) levels in testis homogenate. Fold change vs. controls was obtained as mean ± standard deviation for each experimental group. One-way ANOVA analysis showed significant differences between treated and control rats: * *p* < 0.05 vs. N; ** *p* < 0.01 vs. N; **#** *p* < 0.05 vs. D. Two-way ANOVA analysis for total SOD (A) indicated significant diet effect, extremely significant DDE effect, and no significant interaction. Two-way ANOVA analysis for GPx (B) indicated no significant diet effect, extremely significant DDE effect, and extremely significant interaction (Bonferroni Post-hoc test: *p* < 0.001 D vs. N, *p* < 0.01 D + DDE vs. N + DDE).

With regard to SOD evaluation, the results demonstrated significant decreases in enzymatic activity in D + DDE vs. D (0.66 ± 0.14) and in N + DDE vs. N (0.57 ± 0.14) and D (0.55 ± 0.13). No variation of enzymatic activity was observed in D vs. N and between DDE-treated animals (Figure 2A).

Regarding GPx, a significant decrease of enzymatic activity was measured in D vs. N (0.77 ± 0.08) and in N + DDE vs. N (0.56 ± 0.07) and D (0.72 ± 0.09). In D + DDE, a significant decrease was found vs. D (0.79 ± 0.06). No significant variation of enzymatic activity was detected comparing DDE-treated animal groups (Figure 2B).

### 3.3. High-Fat Diet and DDE Induce Pro-Apoptotic Stimuli

**Figure 3:**
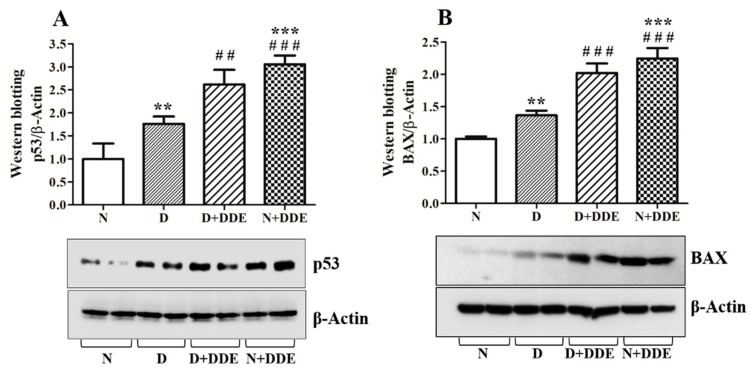
Analysis of p53 (**A**) and BAX (**B**) protein levels in the total homogenate. The data, obtained as mean ± standard deviation, were showed as folds of change vs. N and graphically represented. One-way ANOVA analysis showed significant differences between treated and control rats: ** *p* < 0.01 vs. N; *** *p* < 0.001 vs. N; # # *p* < 0.01 vs. D; # # # *p* < 0.001 vs. D. Two-way ANOVA analysis for p53 (A) indicated no significant diet effect, extremely significant DDE effect, and extremely significant interaction (Bonferroni Post-hoc test: *p* < 0.01 D vs. N). Two-way ANOVA analysis for BAX (B) indicated not quite significant diet effect, extremely significant DDE effect, and extremely significant interaction (Bonferroni Post-hoc test: *p* < 0.001 D vs. N, *p* < 0.01 D + DDE vs. N + DDE).

Western blotting analysis performed on total testis homogenate, showed increases in p53 and BAX protein levels due to the administration of fatty acids and/or pesticide (Figure 3).

D group exhibited a significant increase in p53 levels vs. N (1.73 ± 0.16) (Figure 3A). In DDE-treated animals, p53 levels were the highest among the groups. In D + DDE, a significant increase was detected vs. D (1.48 ± 0.18). In the N + DDE group, a significant increase of p53 was detected vs. N (3.05 ± 0.19) and D (1.73 ± 0.11). No significant changes in the protein levels were found between DDE-treated animals (Figure 3A). Two-way ANOVA analysis suggested an extremely significant DDE effect, with effect of diet only in absence of DDE (D vs. N) confirming no significant variation between D + DDE and N + DDE group.

A similar trend was detected for the pro-apoptotic protein BAX (Figure 3B). In D group, we found a significant increase vs. N (1.36 ± 0.07). In D + DDE, BAX protein levels were found significantly increased vs. D (1.48 ± 0.11). Finally, in N + DDE, BAX levels were found increased vs. N (2.24 ± 0.16) and D (1.64 ± 0.12). No significant variation was detected between D + DDE and N + DDE groups.

**Figure 4:**
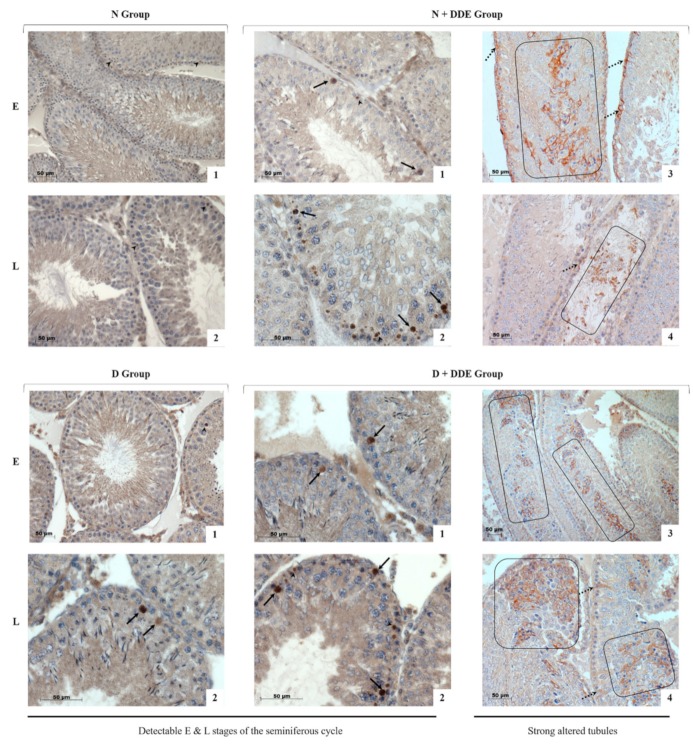
Immunohistochemical stain for cl-Casp3 was faint in the control rats (N group), only some apoptotic residual body were positive (arrowheads, panels 1–2). In D group, an increased number of residual bodies were observed (arrowhead, panel 1), as well as some spermatogonia at basement membrane (black arrows, panel 2). In DDE-treated groups, a larger amount of residual bodies was found in both N + DDE (arrowheads, panel 1) and D + DDE groups (arrowheads, panel 2). In addition, a lot of basal cells in some tubules (spermatogonia and spermatocytes, black arrows) were found positive to cl-Casp3 antibody (N + DDE, panels 1–2); (D + DDE, panels 1–2). The cells depleted in the lumen of the tubules were heavily marked (N + DDE, black rectangle, panels 3–4); (D + DDE, black rectangles, panels 3–4). Moreover, Sertoli cells positive to cl-Cas3 were found in both N + DDE (dashed arrows, panels 3–4) and D + DDE (dashed arrows, panel 4) groups.

To detect apoptotic cells, histological sections were immunostained for the cleaved caspase 3 (cl-Casp3), the main player of the execution phase of apoptosis, to compare the extent of apoptosis in control and treated rats (Figure 4).

In N group, few residual apoptotic bodies were immunostained for cl-Casp3 in the Sertoli cell cytosol (Figure 4, panels 1–2).

In D group, more residual apoptotic bodies were observed together with some positive spermatogonia/dark pachytene spermatocytes (Figure 4, panels 1–2).

Finally, in testis of rats belonging to DDE-treated groups, an increased number of apoptotic bodies was present. cl-Casp3 immunoreactivity was also found in some spermatogonia, spermatocytes (Figure 4, panels 1–2, D + DDE and N + DDE), as well as in immature/degenerating germ cells released in tubular lumina (Figure 4, panels 3–4, D + DDE and N + DDE). Moreover, some labeled Sertoli cells were found in the presence of the pesticide (Figure 4, panels 3–4, D + DDE and N + DDE).

### 3.4. High-Fat Diet and DDE Induce PCNA Up-Regulation

**Figure 5:**
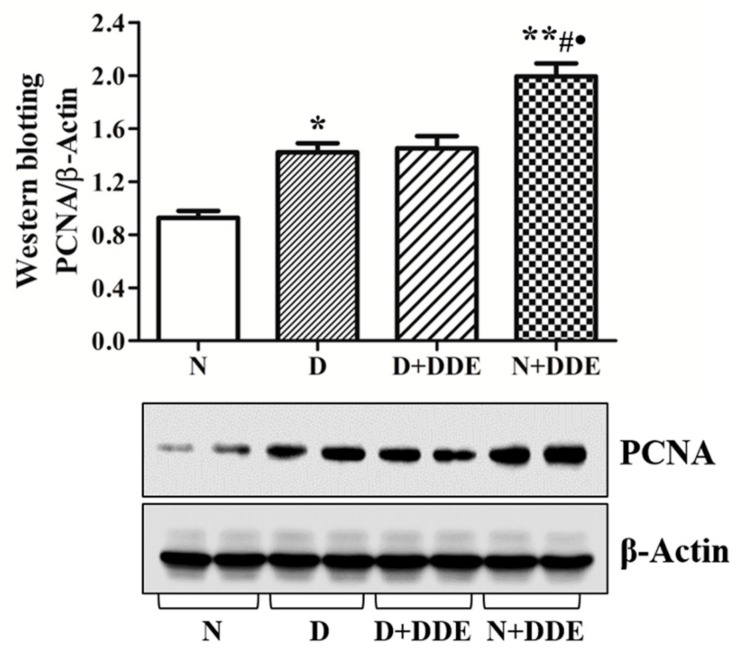
Analysis of proliferating cell nuclear antigen (PCNA) content in testis homogenate. The results obtained as mean ± standard error, were represented as fold of change vs. controls. One-way ANOVA analysis showed significant differences between treated and controls rats:* *p* < 0.05 vs. N; ** *p* < 0.01 vs. N; **#** *p* < 0.05 vs. D; **•** *p* < 0.05 vs. D + DDE. Two-way ANOVA analysis for PCNA indicated no significant diet effect, an extremely significant DDE effect, and an extremely significant interaction (Bonferroni Post-hoc test: *p* < 0.001 D vs. N, *p* < 0.001 D + DDE vs. N + DDE).

Pro-apoptotic effect of fatty acids and DDE could be counterbalanced by cell survival and proliferation monitored by using PCNA protein levels (Figure 5). The results of western blot showed that PCNA content was higher in testis of treated rats vs. N. The levels of PCNA were significantly increased in D group vs. N (1.47 ± 0.12). The highest amount of PCNA was detected in N + DDE group vs. N (2.06 ± 0.16), vs. D (1.41 ± 0.11) and D + DDE (1.40 ± 0.10). Two-way ANOVA showed an extremely significant DDE effect and the Bonferroni Post-hoc test showed a significant difference both in D vs. N and D + DDE vs. N + DDE.

### 3.5. Changes of Metallothioneins Expression and Synthesis

**Figure 6:**
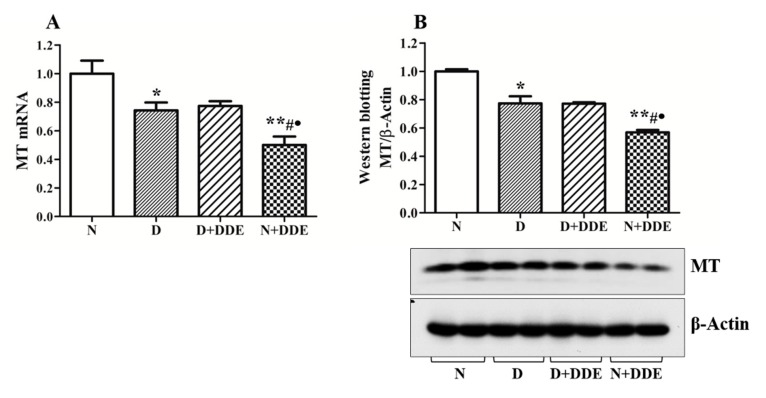
Analysis of metallothioneins (MT) gene expression (**A**) and MT protein levels (**B**) were graphically represented as fold change vs. N. The data were calculated as media ± standard deviation. One-way ANOVA analysis showed significant differences between treated and control rats: * *p* < 0.05 vs. N; ** *p* < 0.01 vs. N; **#** *p* < 0.05 vs. D; • *p* < 0.05 vs. D + DDE. Two-way ANOVA analysis for MT mRNA (A) indicated no significant diet effect, extremely significant DDE effect, and extremely significant interaction (Bonferroni Post-hoc test: *p* < 0.001 D vs. N, *p* < 0.001 D + DDE vs. N + DDE). Two-way ANOVA analysis for MT (B) indicated no significant diet effect, extremely significant DDE effect, and extremely significant interaction (Bonferroni Post-hoc test: *p* < 0.001 D vs. N, *p* < 0.001 D + DDE vs. N + DDE).

Real-time PCR analysis demonstrated a down regulation of MT transcripts in all treated groups compared to the control group (Figure 6A).

In D group, MTs mRNA content was decreased vs. N (0.74 ± 0.05). In N+DDE group, MT mRNA levels were decreased vs. N (0.50 ± 0.06), vs. D (0.67 ± 0.04), and D + DDE (0.65 ± 0.05) groups.

Western blotting analysis of MTs protein content in the total homogenate (Figure 6B) showed a corresponding trend with MTs transcript: D vs. N (0.77 ± 0.05); N + DDE vs. N (0.56 ± 0.02); N + DDE vs. D (0.73 ± 0.02); N + DDE vs. D + DDE (0.74 ± 0.04). No changes were found in both MT mRNA and protein levels between D and D + DDE groups (Figure 6A,B).

### 3.6. High-Fat Diet and DDE Induce Alterations in Testis Morphology

**Figure 7:**
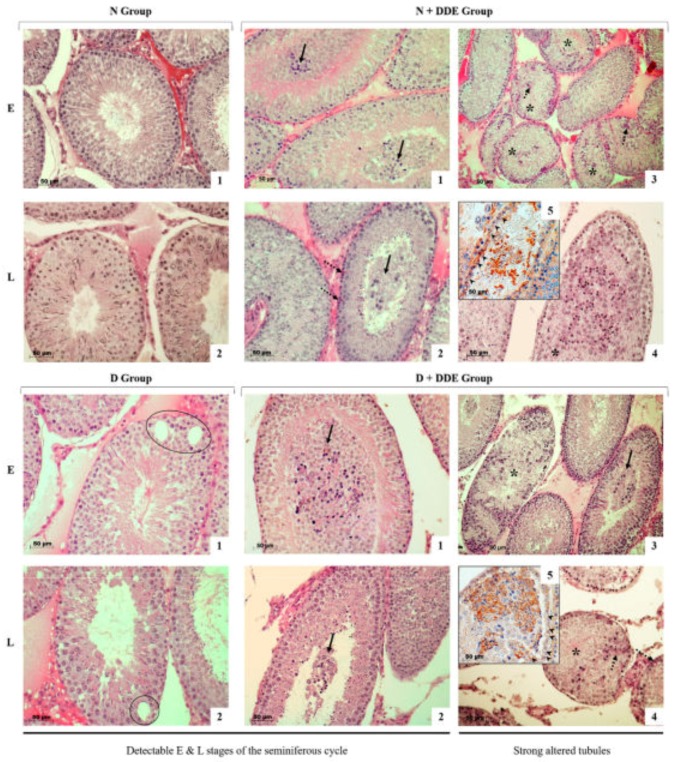
Histological analysis of the tubules of control rats (N group, panels 1–2) showed the sequence of germ cell generation at the stages E (I-VIII) and L (IX-XIV). In D group, some tubules present in the seminiferous epithelium vacuolated and empty areas (panels 1–2, black circles). In DDE-treated rats (N + DDE and D + DDE, panels 1–5), a lot of tubules exhibited the lumen filled with non-differentiated cells mainly round spermatids in E stages along with spermatocytes in L stages (arrows). Severe cellular depletion in some tubules was detected and showed as in the figure for cleaved caspase 3 immunostaining in both N + DDE and D + DDE groups (both panels 5, arrowheads) where, in a predominant way, only Sertoli cells were detectable in the tubular wall. Tubules with a contracted lumen size and strong disorganization of the seminiferous epithelium were found (asterisks). Eosinophilic cells were also detected (dashed arrows). Scale bar 50 µm.

In the testis of control rats (N) the seminiferous tubules showed all stages of the spermatogenic cycle that we divided in early (E, I-VIII stages) and late (L, IX-XIV stages) stages due to 14 specific germ cell associations that are normally detectable [52], (Figure 7, N group, panels 1–2). In rats treated with saturated fatty acids and with DDE, (D, D + DDE, N + DDE), seminiferous tubules exhibited morphological alterations that were more severe in the presence of the pesticide.

In D group, some tubules presented vacuolization and empty areas in the seminiferous epithelium (Figure 7, panels 1–2) and the presence of some eosinophilic cells.

In both DDE-treated groups, the lumina were filled with discharged cells that were prevalently constituted by round spermatids in E stages (Figure 7, panel 1, D + DDE and N + DDE), whereas we found spermatocytes in L stages along with round spermatids (Figure 1, panel 2, D + DDE and N + DDE). In other cases, a contraction of the tubular lumen size could be observed with a strong disorganization of the seminiferous epithelium where it was no longer possible to observe the typical cell associations (Figure 7, panels 3–4, D + DDE and N + DDE). Moreover, in the seminiferous epithelium eosinophilic, germ and Sertoli cells were detected. In some tubules, the depletion of the cells was so severe that their wall was prevalently formed only by Sertoli cells (Figure 7, panel 5, D + DDE and N + DDE).

### 3.7. Serum Testosterone Levels

**Figure 8:**
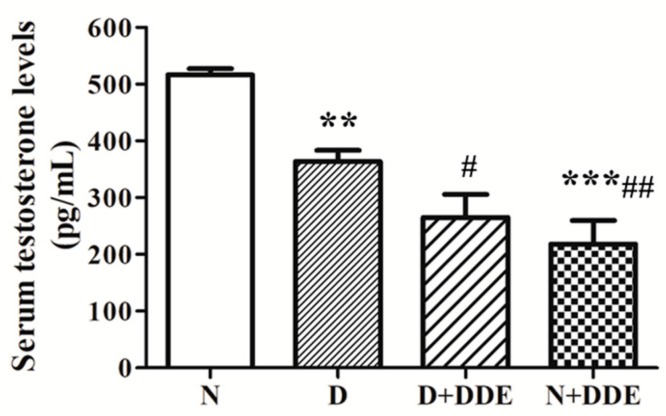
Analysis of serum testosterone levels. The results were graphically represented as pg/mL of testosterone content in rat serum. The data were calculated as media ± standard deviation. One-way ANOVA analysis showed significant differences between treated and controls: ** *p* < 0.01 vs. N; *** *p* < 0.001 vs. N; **#** *p* < 0.05 vs. D; ## *p* < 0.01 vs. D. Two-way ANOVA analysis indicated significant diet effect, extremely significant DDE effect, and extremely significant interaction (Bonferroni Post-hoc test: *p* < 0.001 D vs. N).

Serum testosterone level was monitored in each experimental group. The results showed decreased testosterone levels in D vs. N (0.70 ± 0.02). The highest reduction was found for N + DDE vs. N (0.42 ± 0.07) and D (0.60 ± 0.10). Significative reduction of the testosterone level was also observed in D + DDE vs. D (0.73 ± 0.09). No significant change was evidenced between DDE-treated animals (Figure 8).

### 3.8. Androgen Receptor Content

**Figure 9:**
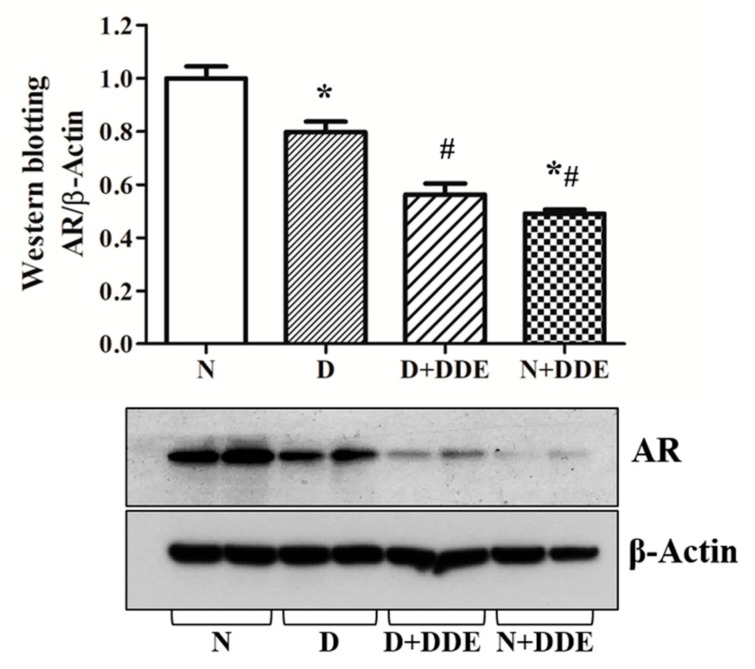
Analysis of androgen receptor (AR) protein levels were graphically represented as fold change vs. N. The data were calculated as media ± standard deviation. One-way ANOVA analysis showed significant differences between treated and controls rats: * *p* < 0.05 vs. N; **#** *p* < 0.05 vs. D. Two-way ANOVA analysis indicated significant diet effect, extremely significant DDE effect, and extremely significant interaction (Bonferroni Post-hoc test: *p* < 0.001 D vs. N).

AR protein content was measured in the total homogenate. Western blotting data showed decreased levels in D vs. N (0.76 ± 0.01). Moreover, DDE-treated animals showed lower AR levels in testis. In fact, in D + DDE, AR was reduced vs. D (0.69 ± 0.02). Finally, in N + DDE group, AR content was decreased vs. N (0.47 ± 0.01) and D (0.63±0.01). No significant variations were detected between DDE-treated animals (Figure 9).

## 4. Discussion

Data herein described indicate that a high-fat diet and chronic oral exposure to low doses of DDE for four weeks affect rat testis function and morphology differently. Our results showed alterations in tissue morphology and metabolism (oxidative stress, lipid peroxidation, apoptosis, and cell proliferation) as well as impairment in endocrine function (decrease in testosterone and AR levels).

In group D, increased levels of MDA vs. control animals were observed, indicating oxidative damage in the lipid component of cells that could be a major factor of further tissular damage. The response of the antioxidant enzymes was differently modulated. We did not find variation in the activity of total SOD in D vs. N group, whereas it was higher in D than in DDE-treated rats. The activity of GPx had decreased in all treated groups vs. N. Moreover, in D group, we found a reduction in MT gene expression and protein content. In fact, MTs are proteins rich in cysteine, carriers of heavy metals, involved in both the detoxification processes and mechanisms of protection against oxidative stress. These MTs were found down regulated in oxidative stress conditions induced by fat [53]. As the consequence of occurred oxidative stress, p53 level, the guardian of the genome, was found to be increased. p53 is led to organize and coordinate the cellular responses behaving in a different way according to the entity of the stress. When the level of oxidative stress is low, p53 exhibits antioxidant activities to eliminate stress conditions and ensure cell survival; when the level of oxidative stress is high, p53 exhibits pro-oxidative activities leading to cell death [54]. p53 regulates the expression of genes, such as BAX, involved in cellular responses to oxidative stress [55]. Accordingly, our data showed increased BAX protein levels and cl-caspase 3 positivity in D group. This finding is in line with previous results showing that the administration of saturated fatty acids in cultured Sertoli cells caused a dose-dependent decrease of cell survival through upregulation of BAX [56]. Normally, testicular apoptosis removes the excess of germ cells (75%) and abnormal spermatozoa during spermatogenesis [57,58], to adapt germ cell population to support the capacity of Sertoli cells [59]. In a non-physiological condition, excess of apoptosis can affect spermatogenesis and lead to infertility [60]. In contrast to these death signals and probably in an attempt to balance them, testis activated cell proliferation by increasing PCNA protein content. Therefore, the increase in PCNA levels found in D group may account for a physiological adaptation to chronic high-fat feeding. Finally, morphological analysis showed many tubules exhibiting histological alterations in the seminiferous epithelium, with the presence of micro and macro-vacuolization due to early damage of Sertoli cells that may contain fluid or lipids or may represent spaces left by the depleted germ cells [61]. Conversely, vacuoles can occur by the swelling of membrane-bound organelles, such as the endoplasmic reticulum and identified as autophagic vacuoles or by endocytosis resulting from invagination of the plasma membrane [36,62]. At a systemic level, our D group showed decreased serum testosterone levels according to Fan and co-workers [63] who found altered protein levels of aromatase that converts testosterone in oestradiol. This drop in testosterone levels seemed to act as a downstream target, the AR. D group showed decreased testis AR content compared to N group, also in line with the results evidenced by other researchers who found declines in the tight junction-related proteins, among the others’ AR, one of the essential contributors of the blood–testis barrier (BTB), disrupting in this way the integrity of BTB and accounting for the observed morphological alterations [63].

In DDE-treated rats, our results indicate that DDE administration caused an increase in MDA levels, testifying oxidative damage in cellular lipid component different in D+DDE and in N+DDE groups. In D+DDE animals, the levels of MDA were significantly increased vs. D, whereas N+DDE group showed the highest values of MDA among the groups. These data suggest that DDE plays a key role in the oxidative stress generation, mostly when it was not associated with a high-fat diet. Probably, in association to fatty acids, a part of DDE, due to its lipophilic nature, remains stored in an inert form in the adipose tissue or in the other body compartments rich in fatty acids inducing less toxicity in terms of oxidative stress generation [64]. In fact, MDA content was progressively higher in D, D + DDE, and N + DDE vs. N group. For this reason, it can be suggested that there was not a synergic effect of fatty acids and pesticide, in accordance with previous results [53,65]. Regarding the described pro-oxidative effects of DDE, in both D + DDE and N + DDE groups, the two anti-oxidant system activities were similarly reduced vs. N and D groups, in accordance with other authors [62]. It can be suggested that, at first, the two antioxidant systems could try to quench ROS accumulation, but in the long term, an impairment of the anti-oxidant systems occurred, probably due to an excessive production of H_2_O_2_ that, in turn, caused an inactivation of both SOD and GPx. An increase in H_2_O_2_ production was, in fact, reported in testis of rats treated with DDT [66]. Moreover, MT levels differently decreased in DDE treated animals: In D + DDE, they did not differ from D but were significantly higher than in N + DDE. For this reason, we assumed that MT levels decreased according to the increase in MDA and to the impairment of the antioxidant system and hence of ROS accumulation [66]. BAX levels increased progressively from N to N + DDE according to p53 content. As the consequence of increased BAX levels, a raise in the number of cleaved caspase 3-positives cells were detected in testis sections. Moreover, mainly in N + DDE group, the increase in PCNA protein content availed to compensate the cell death number, raising in conjunction with oxidative injury (by MDA), to maintain homeostasis of spermatogenesis and fertility. It is interesting to note that pro-apoptotic protein p53 content increased in both DDE treated groups with no significant difference between N + DDE and D + DDE group, whereas the increase in proliferation protein PCNA was greater in N + DDE than in D + DDE group. The excess of PCNA content in N + DDE group compared to the other treated groups may be explained as an adaptive response not only to compensate the increased apoptotic stimuli, but also to further compensate induction of oxidative stress greater than in D groups, as shown by an excessive increase in MDA content. Our data agree with previous evidence [35], where authors found induction of apoptosis through cytosolic and mitochondrial paths. In addition, it has been shown that DDE induced cell proliferation through oxidative stress [67]. Finally, histological observations showed that the pesticide did not generate appreciable variations between D + DDE and N + DDE groups. Damaged tubules essentially showed more severe alteration than in D rats. In fact, many seminiferous tubules showed exfoliation of germ cells in the lumina. This condition is similar to the one that can be found in different hormonal disturbances and following different treatments with toxicants in vivo [68,69,70]. Our findings argued that DDE functions as an endocrine disruptor implicated in the reduction of tissular and serum testosterone levels as well as in the alteration of androgen receptors responses, according to literature data [71,72].

## 5. Conclusions

In conclusion, our results demonstrated that the excess of saturated fatty acid intake, as well as the administration of a low dose of DDE gave rise to the onset of molecular and morphological alterations in rat testis, more pronounced for the pesticide. The damage from fatty acids and DDE seems mainly due to oxidative stress and testosterone levels that maintain spermatogenic homeostasis by inhibiting death signals of germ cells on one side [73] and AR inhibition on the other side [74,75]

In our experimental conditions, we can observe a certain degree of adaptation to counterbalance the adverse effects of fatty acids and DDE in terms of increased cellular proliferation. Using this strategy, the testicular cells can generate opposite paths to maintain a pool of seminiferous tubules composed of functional cells capable of undergoing a normal differentiation and maturation to sustain spermatogenesis and fertility.
